# Peak longitudinal strain most accurately reflects myocardial segmental viability following acute myocardial infarction - an experimental study in open-chest pigs

**DOI:** 10.1186/1476-7120-10-23

**Published:** 2012-05-29

**Authors:** Erling Aarsæther, Assami Rösner, Espen Straumbotn, Rolf Busund

**Affiliations:** 1 Department of Cardiothoracic and Vascular Surgery, University Hospital of North Norway, N-9038, Tromsø, Norway; 2 Institute of Clinical Medicine, University of Tromsø, N-9037, Tromsø, Norway

**Keywords:** Acute myocardial infarction, Pigs, Speckle tracking echocardiography

## Abstract

**Background:**

The extension and the transmurality of the myocardial infarction are of high predictive value for clinical outcome. The aim of the study was to characterize the ability of longitudinal, circumferential and radial strain measured by 2-dimensional speckle tracking echocardiography (2D-STE) to predict the extent of necrosis in myocardial segments following acute myocardial infarction and to separate transmural necrotic segments from non-transmural necrotic segments in a full 18-segment porcine model.

**Methods:**

2D-STE strain was assessed in long- and short-axis following myocardial infarction in ten open-chest anesthetized pigs. Strain was defined according to systolic peak values. In segments displaying both negative and positive peaks, only the peak with the highest absolute value was utilized. Necrosis was measured by 2,3,5-triphenyltetrazolium chloride (TTC) staining and expressed as percent of each myocardial segment.

**Results:**

Significant correlations were found between the extension of necrosis and all measured parameters of myocardial deformation (*p* < 0.001), but was stronger for longitudinal strain (r^2^ = 0.52) than circumferential strain (r^2^ = 0.38) and radial strain (r^2^ = 0.23). The area under the receiver operator characteristic curve (AUC) for separating transmural necrotic segments (>50% necrosis) from predominantly viable segments (0–50% necrosis) was significantly larger for longitudinal strain (AUC = 0.98, CI = 0.97–1.00) when compared with circumferential strain (AUC = 0.91, CI = 0.84–0.97, *p* < 0.05) and radial strain (AUC = 0.90, CI = 0.83 – 0.96, *p* < 0.01), indicating a stronger ability of longitudinal strain to identify segments with transmural necrosis.

**Conclusion:**

Peak strain values derived from 2D-STE correlate well with the extent of necrosis in myocardial segments following acute myocardial infarction. Longitudinal strain most accurately reflects myocardial segmental viability in this setting.

## Background

Two-dimensional speckle tracking echocardiography (2D-STE) is an increasingly recognized technique that measures myocardial deformation by automatic tracking of interference patterns from conventional gray scale B-mode images during the cardiac cycle. In contrast to tissue Doppler derived strain, 2D-STE has the advantage of being relatively independent of insonation angle, and can therefore assess motion in three dimensions [1,2]. 2D-STE has been validated against sonomicrometry in experimental studies of myocardial ischemia [2,3] and magnetic resonance imaging in patients with ischemic heart disease and the results have been encouraging [4,5].

The extent of the necrotic myocardium following myocardial infarction (MI) has prognostic as well as therapeutic implications [6]. Traditionally, the presence of ST-elevation in the electrocardiogram has been used as a criterion for selection of patients with extensive myocardial damage who should be scheduled for urgent coronary angiography and revascularization. However, 30% of patients with acute coronary occlusion do not develop ST-segment elevation, but may still suffer substantial myocardial necrosis [7]. 2D-STE is a non-invasive method that might be utilized in the acute clinical setting to detect the extent and transmurality of infarctions and hence to select patients with non-ST-elevation MIs who should be scheduled for urgent revascularization. Experimental studies in rodents have shown that the orthogonal strain components derived from short axis images, i.e. circumferential strain and radial strain, are strongly correlated to both left ventricular scarring and necrosis following MI [8,9]. Chan et al. examined short-axis derived strain parameters as well as longitudinal strain in patients with chronic ischemic left ventricular dysfunction. According to the study by Chan et al., longitudinal strain distinguished subendocardial necrosis from late-enhancement negative myocardium, but in contrast to circumferential strain, longitudinal strain was unable to discriminate between subendocardial and transmural necrosis [10]. Sjøli et al. demonstrated in a clinical study involving patients with first time MI that circumferential strain better separated between subendocardial necrosis and transmural necrosis on a segmental level than longitudinal strain [11]. However, none of these studies included apical or basal short-axis images. The aim of the present study was therefore to examine the ability of longitudinal strain, circumferential strain and radial strain to separate transmural necrotic segments from non-transmural necrotic segments in a full 18-segment porcine model with short axis-images from the base, middle and apical parts of the left ventricle following acute MI.

## Methods

### Protocol

The experimental protocol was approved by the local steering committee of the Norwegian Animal Experiments Authority. Animal care was done in accordance with guidelines from the U.S. National Institute of Health (NIH Publication No. 85–23, revised 1996). Ten pigs fasted overnight, but with free access to water were premedicated with im. ketamine (20 mg/kg, Warner Lambert Nordic, Sweden) and atropine (2 mg/kg, Nycomed Pharma, Norway) and transferred to the operating room. After iv. bolus injections of fentanyl (Pharmalink, Sweden) and pentobarbital (Nycomed Pharma), the pigs were tracheostomized and ventilated on a respirator (Servo Ventilator 900D, Siemens, Sweden). Tidal volume was controlled by repeated blood gas analysis, and pCO_2_ was kept between 4 and 6 kPa. Continous anesthesia with midazolam 0.3 mg/Kg/hour (Alpharma, Norway), fentanyl 0.02 mg/kg/hour (Pharmalink) and pentobarbital 4 mg/Kg/hour (Nycomed Pharma) was established through a central venous catheter in the left jugular vein. The pigs received amiodarone as arrhythmia prophylaxis and 2500 IU of heparin to prevent from clotting of vascular catheters. Blood pressure was measured in the descending aorta with a polyethylene catheter through the right femoral artery and the bladder was drained through a cystostoma. The pigs received 10 ml/Kg/hour glucose enriched sodium chloride (1.25 g glucose/l sodium chloride) as basal fluid replacement. The heart was exposed through a median sternotomy. The pericardium was opened and a flow probe was placed snugly around the pulmonary trunk for continuous measurements of cardiac output. After baseline measurements of basic hemodynamics, MI was induced by placing a small metal clamp on the left anterior descending coronary artery (LAD) 6 cm distal to the division of the left main coronary artery. The position of the LAD clamp was determined from pilot experiments, which indicated that a substantial number of pigs developed ventricular fibrillation during ischemia when the LAD was clamped within a shorter distance from the division of the left main coronary artery. After one hour of ischemia, the clamp was removed and the infarcted area was reperfused for three hours.

### Two-dimensional speckle tracking echocardiography

**Figure 1:**
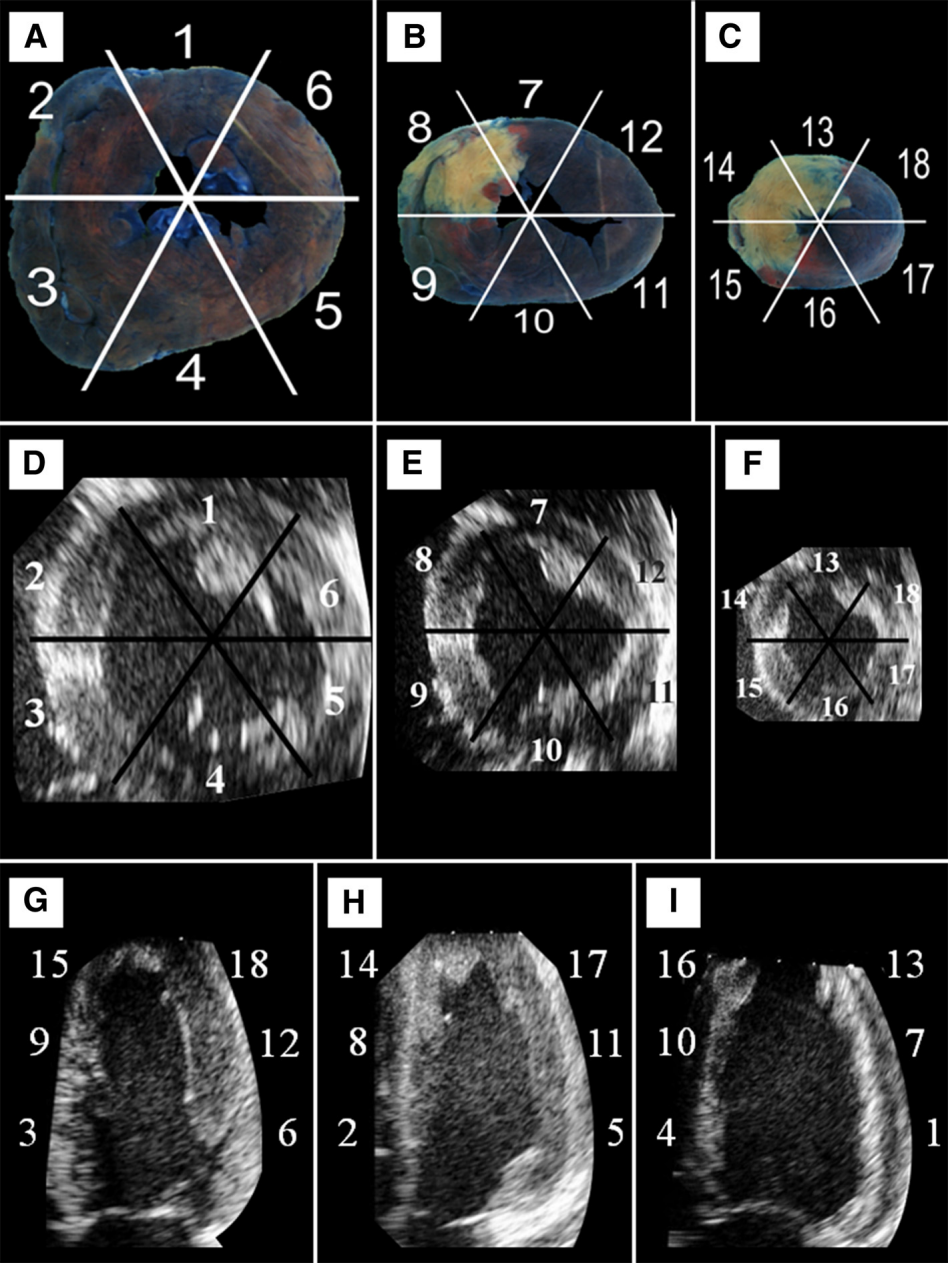
Representative cross sections of porcine hearts stained with Evan’s blue and TTC following myocardial infarction (A–C) and the corresponding areas of the left ventricle depicted by echocardiography in short-axis (D–F) and long-axis (G–I). 1, 7, 13 = anterior; 2, 8, 14 = antero-septum; 3, 9, 15 = inferoseptum; 4, 10, 16 = inferior; 5, 11, 17 = infero-lateral; 6, 12, 18 = antero-lateral.

**Figure 2:**
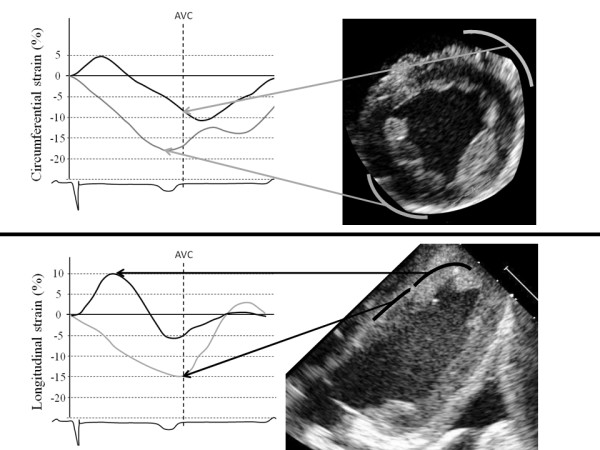
**Representative strain curves following MI derived from circumferential strain (top) and longitudinal strain analysis (bottom).** The infarcted apical anteroseptum exhibits biphasic strain curves (black) following circumferential and longitudinal strain analysis. In contrast to peak circumferential strain, peak longitudinal strain is a positive strain value. AVC = Aortic valve closure.

All images were obtained after opening of the pericardium and the recordings were performed at baseline and following 3 hours of reperfusion. The thoracic cavity was filled with 38°C of saline, in order to provide air-free insonation. The left ventricle was visualized by basal, middle and apical short axis images as well as long axis images in 2-, 3- and 4-chamber views (Figure 1). A Philips iE33-ultrasound-machine (Philips Medical Systems, Andover, MA, U.S.) was used with a 5 MHz transesophageal probe that was positioned epicardially. Apical, middle and basal short axis views were taken from the right ventricular side in order to depict the left ventricle at a lower sector-angle. Apical views were taken also from an apical right ventricular approach for 4- and 3-chamber views, while imaging loops of the anterior and posterior wall were acquired positioning the probe towards the posterior apical and anterior apical segment respectively. By reducing imaging depth and insonation angle without reducing spatial resolution, the technique provided images with a frame rate of 78 ± 11 Hz. All images were analyzed off-line by dedicated software (Syngo® Velocity Vector Imaging, Siemens, Erlangen, Germany). Three cardiac cycles were acquired and averaged by the software. The correct tracking of border-zones was visually controlled and manually corrected. Ejection time was defined by Doppler-registered aortic valve opening and closure. Quality was assessed on 2D images only. Peak positive or peak negative strain was extracted from each strain curve. For segments displaying biphasic strain curves, i.e. strain curves with both positive and negative peaks (Figure 2), only the peak with the highest absolute value was utilized, irrespective of sign.

### Staining of the hearts

After three hours of reperfusion, the pigs received bolus injections of pentobarbital. The aorta was clamped and the hearts were perfused with 3 L of saline through a cannulla in the ascending aorta to wash out the blood from the myocardium, which would otherwise interfere with the tetrazolium staining [12]. After saline perfusion, the LAD clamp was replaced and Evans blue was injected into the ascending aorta to separate the non-at-risk area from the risk area. The hearts were cut out and frozen overnight. After 12 hours of freezing, the hearts were thawed and cut in six 1 cm slices from apex to base parallel to the atrioventricular groove. The slices were incubated for 15 minutes in 2,3,5-triphenyltetrazolium chloride (TTC) at 37°C followed by bleaching in formalin for 30 minutes. The slices were fixed between two glass sheets and non-at-risk area, the area at risk and the necrotic area were determined by planimetry. The basal side of the slices was measured for technical purposes, thus avoiding measurements of the apical side of the apex, where it was difficult to distinguish between myocardium stained by Evans blue and TTC. Scanned drawings of the slices were geometrically divided into an 18-segment model using the papillary muscles and the anterior and inferior insertion of the right ventricle to the left ventricle as markers, with six segments at each left ventricular level [13]. Apical, middle and basal necrosis was defined as the sum of the necrosis measured in the two apical, two middle and two basal slices respectively. The segments were divided into the following groups on the background of the distribution of necrotic myocardium: transmural necrotic (necrosis > 50%), subendocardial necrotic (1–50% necrosis) and viable (necrosis = 0). The combination of the two latter groups is referred to as predominantly viable (0–50%).

### Variability analysis

To estimate intra- and interobserver variability, the full 18-segment model was re-analyzed for longitudinal, circumferential and radial strain in five randomly chosen pigs.

Variability was assessed by intraclass correlation coefficients. Intraclass correlation coefficients were analyzed with SPSS (Chicago, IL, U.S.) using two-way random single measures with absolute agreement according to intra class correlation coefficient 2.1 by Shrout and Fleiss [14]. Intra-observer variability was determined by comparison of analyses recalculated by the same observer after a minimum of four weeks. Interobserver variability was obtained by comparing analysis of the same segments from identical images by two different observers. The intraclass correlation coefficient was considered fair when between 0.4 and 0.59, good when between 0.60 and 0.74 and excellent when ≥ 0.75.

### Statistical methods

Data are presented as mean ± standard deviation, unless otherwise noted. Differences in hemodynamic variables between baseline and following ischemia and reperfusion were analyzed with paired samples *t* tests. The relationship between necrosis and strain was assessed by linear regression. Comparison of the number of segments exhibiting positive strain values between longitudinal and short-axis derived parameters of myocardial deformation were analyzed with Fisher’s exact test. Comparisons of strain values obtained from segments at baseline, and in viable, subendocardial and transmural necrotic segments following MI were analyzed with one way analysis of variance and the Bonferroni posthoc test. The optimal cut off values for longitudinal, circumferential and radial strain were determined from the receiver operator characteristic (ROC) curve by choosing values that combined the highest sensitivity with the highest possible specificity. Diagnostic accuracy was defined as true negative and true positive segments divided by all segments. Comparison of ROC curves was performed with the Medcalc 11.1 statistical program (MedCalc, Gent, Belgium) according to the method described by *DeLong et al.*[15]. All other statistical analysis was performed with the SPSS 16.0 statistical software program (Chicago, IL, U.S.).

## Results

### Basic hemodynamics

Basic hemodynamic data, ejection fraction, end diastolic volume and end systolic volume at baseline and following ischemia and three hours of reperfusion are summarized in Table 1. Mean arterial pressure, cardiac output, stroke volume and end diastolic volume dropped significantly following ischemia and reperfusion, whereas a significant increase was found with respect to heart rate and central venous pressure. Mean ejection fraction and end systolic volume dropped following ischemia and reperfusion, but the difference failed to reach statistical significance.

**Table 1:** Basic hemodynamic data, ejection fraction, end diastolic volume and end systolic volume for ten pigs at baseline and following one hour of regional ischemia and three hours of reperfusion

**Index**	**Baseline**	**Following ischemia** **and reperfusion**
MAP (mm Hg)	94.5 ± 5.5	77.2 ± 13.7*
HR (beat/min)	86.8 ± 9.7	148.8 ± 18.3#
CO (l/min)	3.6 ± 0.5	2.9 ± 0.5*
SV (ml)	42.6 ± 8.1	19.8 ± 4.3#
CVP (mmHg)	6.3 ± 1.7	7.9 ± 1.6§
EF (%)	56.4 ± 8.3	45.8 ± 13.5
EDV (ml)	82.0 ± 17.0	53.4 ± 16.2§
ESV (ml)	36.2 ± 10.5	28.1 ± 7.5

Data are presented as mean ± standard deviation. *p < 0.05, §p < 0.01, #p < 0.001 vs baseline. CO = Cardiac output, CVP = Central venous pressure, EF = Ejection fraction, EDV = End diastolic volume, ESV = End systolic volume, HR = Heart rate, MAP = mean arterial pressure, SV = Stroke volume.

### Necrosis

**Figure 3:**
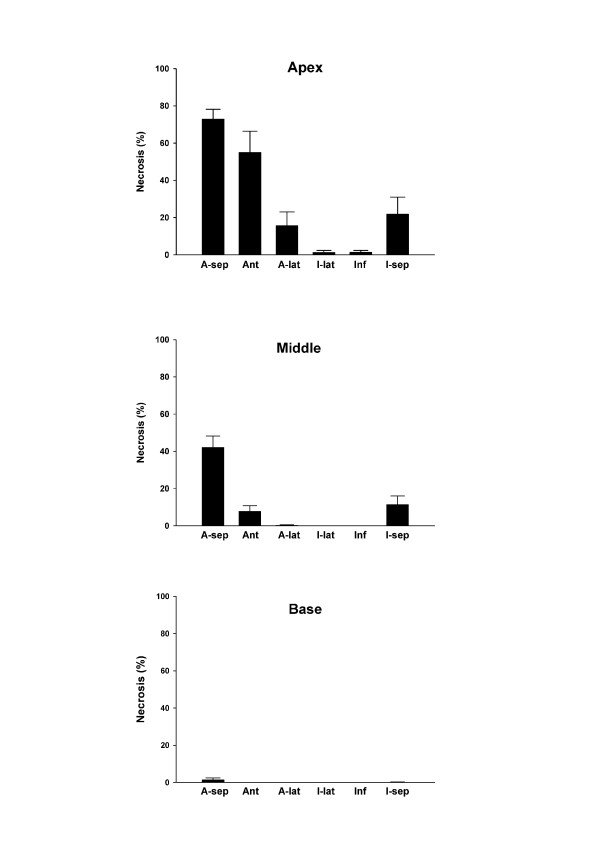
**Distribution of necrosis in each of the 18 myocardial segments in the apex (top), middle (middle) and base (bottom).** A-lat = antero-lateral; Ant = anterior; A-sep = antero-septum; I- lat = infero-lateral; Inf = inferior; I-sep = infero-septum.

Representative cross sections of the heart following staining with Evan’s blue and TTC are demonstrated in Figure 1A–C. The necrosis was exclusively confined to midventricle and the apex (Figure 3). Necrosis comprised 10.1% ± 3.2 of the left ventricle and was most pronounced in the antero-septal (73% ± 16) and anterior (55% ± 36) parts of the apical sections.

### Relationship between necrosis and strain

Highly significant correlations were found between necrosis and longitudinal, circumferential and radial strain respectively (Table 2). The strongest correlation was found between necrosis and longitudinal strain (r^2^ = 0.52), followed by circumferential strain (r^2^ = 0.38) and radial strain (r^2^ = 0.23).

**Table 2:** Correlations between strain and the extension of necrosis in 180 segments following acute myocardial infarction in pigs

**Index**	**r**^**2**^	***p***
Longitudinal strain	0.52	<0.001
Circumferential strain	0.38	<0.001
Radial strain	0.23	<0.001

### Myocardial deformation parameters at baseline and following MI in viable, subendocardial and transmural necrotic segments

**Figure 4:**
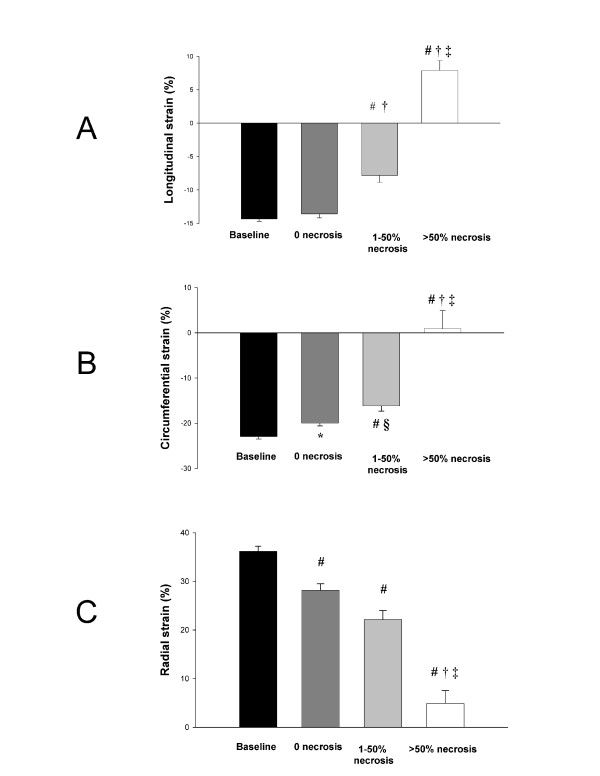
**Longitudinal strain (A), circumferential strain (B) and radial strain (C) in baseline segments (black), in segments without necrosis following MI (dark gray), segments with 1–50% necrosis (gray) and in segments with more than 50% necrosis (white).** #*p* < 0.001 vs. baseline, †*p* < 0.001 vs. 0 necrosis, ‡*p* < 0.001 vs. 1–50% necrosis, *p < 0.01 vs. baseline, §*p* < 0.05 vs. 0 necrosis.

VPeak strain values obtained at baseline and following MI from long- and short-axis images are displayed in Figure 4. No significant differences were found between longitudinal strain at baseline and longitudinal strain in viable segments following MI. In contrast, viable segments following MI exhibited significant lower circumferential strain and radial strain when compared with baseline. Subendocardial necrotic segments exhibited significantly lower strain when compared to baseline strain for all examined myocardial deformation parameters. Longitudinal strain and circumferential strain, but not radial strain separated subendocardial necrotic segments from viable on a significant level. For all three dimensions of strain, transmural necrotic segments were significantly different from subendocardial necrotic as well as viable segments.

### Transmural necrotic segments exhibited biphasic strain curves

Representative strain curves from viable (gray) and transmural necrotic myocardial segments (black) are displayed in Figure 2. As illustrated in this figure, transmural necrotic segments displayed a biphasic pattern, in which both negative and positive peaks are frequently observed. Strain is defined as (L_(t)_ –L_0_)/L_0_, in which L_0_ represents the end diastolic distance between to pixels and L_(t)_ the longest distance between these two pixels during systole. Accordingly negative strain values indicate shortening of the myocardium, which is normally seen in the circumferential and longitudinal projections. In contrast, there is a thickening of the myocardial wall in the radial direction, which results in positive radial strain under normal conditions. The positive peaks displayed by the transmural necrotic segments in Figure 2 typically represent abnormal lengthening of the myocardium during systole.

### Peak longitudinal strain more often detected abnormal systolic lengthening following MI

As illustrated in Figure 2, the first part of the biphasic strain curve, i.e. the one that represents the abnormal systolic lengthening, was often more pronounced in transmural necrotic segments analyzed by longitudinal images than by images recorded in the short-axis, leading to more positive peak strain values following longitudinal strain analysis. Positive longitudinal strain was found in 15 transmural necrotic segments, indicating stretching of the tissue during systole. In contrast, positive circumferential strain and negative radial strain, indicating stretching of the tissue during systole were only found in six transmural necrotic segments (p < 0.01 compared with longitudinal strain).

### Diagnostic accuracy

**Figure 5:**
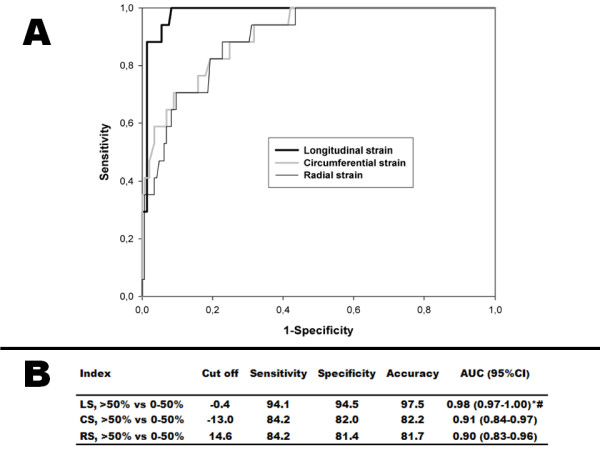
**A. ROC-curve for longitudinal strain, circumferential strain and radial strain to discriminate between predominantly viable segments (0–50% necrosis) and transmural necrotic segments (>50% necrosis).** The area under the curve is significantly larger for longitudinal strain when compared to circumferential strain (*p* < 0.05) and radial strain (*p* < 0.01). **B.** ROC curve analysis for strain parameters to separate transmural necrotic segments (>50% necrosis) from predominantly viable (0–50% necrosis). AUC = Area under the curve; CI = Confidence interval; CS = Circumferential strain; LS = Longitudinal strain; RS = Radial strain. *p < 0.05 versus circumferential strain. #*p* < 0.01 versus radial strain.

Diagnostic accuracies for these myocardial deformation parameters to discriminate between transmural necrocis and predominantly viable segments were assessed by ROC curves. The corresponding cut off values, sensitivities, specificities and diagnostic accuracies are summarized in Figure 5. Longitudinal strain had the highest diagnostic accuracy to separate transmural necrotic from predominantly viable myocardial segments (97.5%, Figure 5). The area under the ROC curve indicated that peak longitudinal strain separated transmural necrotic segments from predominantly viable myocardial segments significantly better than circumferential strain (*p* < 0.05) and radial strain (*p* < 0.01). Longitudinal strain also had the highest diagnostic accuracy to separate transmural necrotic segments from subendocardial necrotic segments, but the area under the ROC curve failed to reach statistical significance when compared with circumferential strain and radial strain (data not shown).

### Inter- and intra-observer variability

Results from inter- and intraobserver variability analysis are summarized in Table 3. The intraclass correlation coefficients indicated excellent reproducibility for all parameters of myocardial deformation.

**Table 3:** Inter- and intra-observer variability

**Index**	**Inter-observer** **variability**	**Intra-observer** **variability**
Longitudinal strain	0.76 (0.65–0.84)	0.89 (0.83–0.93)
Circumferential strain	0.77 (0.66–0.84)	0.75 (0.63–0.83)
Radial strain	0.82 (0.72–0.88)	0.86 (0.79–0.91)

Inter- and intra-observer variability as determined by intraclass correlation coefficients with 95% confidence intervals.

## Discussion

The present study is to our knowledge the first that has compared circumferential strain, radial strain and longitudinal strain with respect to the extension of necrosis in acute ischemia in a complete model, covering all segments of the left ventricle. Our results indicate that peak longitudinal strain better separates transmural necrotic segments from predominantly viable segments compared with circumferential strain and radial strain. Chan et al. have previously examined the diagnostic accuracy of circumferential strain and longitudinal strain in patients with chronic ischemic ventricular dysfunction. In that study it was demonstrated that circumferential strain was able to differentiate transmural and subendocardial necrotic segments as well as transmural necrotic and normal myocardial segments [10]. Furthermore, it was shown that longitudinal strain discriminated between normal and subendocardial necrotic segments, but not between subendocardial and transmural necrotic segments. The high sensitivity of longitudinal strain to detect functional impairment due to subtle ischemia could be explained by the predominant longitudinal orientation of subendocardial fibres, which are more susceptible to ischemia [16,17].

In contrast to the study by Chan et al., the present study has utilized peak positive or negative systolic strain values in acute ischemia, which resulted in positive longitudinal strain values in a substantial number of the transmural necrotic segments, indicating systolic stretching of the myocardium. Positive peak longitudinal strain has previously been demonstrated in necrotic myocardium from dogs subjected to occlusion and reperfusion [18] and was reported in 16 of 17 patients following LAD occlusion during angioplasty [19]. Positive strain to assess myocardial viability has also been used previously [11,20-22]. Gjesdal and colleagues examined the ability of longitudinal, circumferential and radial strain to identify global infarct mass ≥ 30 g in patients with ST-elevation MIs [21]. Using ROC analysis, they found that longitudinal strain by 2D-STE revealed the highest sensitivity and specificity for detection of transmural segmental MI. But in contrast to the present study, the measurement of strain and infarction mass in the work by Gjesdal and colleagues was undertaken a mean of 8.5 months following index ischemia. Although their results may resemble those of the present study, the mean longitudinal strain values are much lower than those of the present study, which probably reflects profound differences in contractile and elastic properties between scarred myocardial tissue and acute ischemia.

In contrast to the present study, Sjøli et al. utilized peak strain in acute ischemia to demonstrate that circumferential strain separated transmural from subendocardial necrosis better than longitudinal strain on a segmental level. It is noticeable that the mean longitudinal strain values from transmural necrotic segments in the study by Sjøli’s group are considerably lower than those of the present study. As illustrated in Figure 2, the results from the analysis of biphasic strain curves are highly dependent on whether the strain values are extracted from the positive peak or from the subsequent negative peak. The mean longitudinal strain value from transmural necrotic segments in the study by Sjøli’s group, resemble those from the present study when only negative peaks were extracted from the strain curves (data not shown), indicating that the number of positive strains chosen for analysis probably were much lower than in our study. In Figure 2, the black curve in the top of the figure indicates that circumferential strain in the transmural necrotic segment is found to display a slightly higher negative value than longitudinal strain, represented by the black curve in the bottom of the figure. Although both strain curves share a common biphasic pattern, the bottom panel shows that peak longitudinal strain is positive, resulting in much higher differences between peak values of predominantly viable and transmurally necrotic tissue compared to circumferential strain. Thus, when peak negative strain was used, instead of peak systolic strain in the present study, the difference between longitudinal strain and circumferential strain to separate transmural necrotic from predominantly viable segments was lost. We assume that Sjøli’s group found slightly higher negative peaks or chose only positive peaks for analysis when negative peaks were not present. The ischemic time and the length of reperfusion time is also of importance, since longitudinal strain has been shown to reach a maximum positive peak following 15 minutes of ischemia, gradually dropping following ischemia beyond 15 minutes and also following reperfusion [18]. The time from thrombolytic therapy to echocardiographic examination in the study by Sjøli’s group was similar to that of the reperfusion time in the present study. However, the mean ischemic time was 163 minutes compared to only 60 minutes in the present study. We therefore suggest that the relatively longer ischemic time in the work by Sjøli et al. may contribute to more dominant negative peaks, and hence lower mean longitudinal strain values than those of the present study.

Another theoretical assumption for finding differences between humans and pigs could be that the porcine left ventricle has different myocardial fiber orientation compared to humans. However, studies of human and porcine myocardial architecture have reported that they are essentially similar [23-27]. Furthermore the size of the MI was larger in the study by Sjøli’s group compared to the present study (15% ± 11 versus 10% ± 3). Larger MIs probably involve more segments in the mid-ventricular short axis, where circumferential strain might be most affected. In the present study, the largest infarct zones were present in the apical segments, where the mid-myocardial layers change from circumferential to an oblique alignment. Thus, necrosis in the apex might affect longitudinal motion more than in the mid-ventricular parts.

Positive longitudinal strain and negative circumferential strain reported from an identical myocardial segment might seem as a contradiction to the incompressibility of the myocardial tissue. However, as previously demonstrated segments with acute transmural necrosis exhibited biphasic strain curves in all three dimensions, indicating tissue stretch in the first part of systole and a contraction like deformation in opposite direction in the end of systole. Thus, peak positive and negative systolic strain in the three dimensions was measured at different time points during ejection time period. The abnormal systolic longitudinal lengthening (positive strain) seems as an accurate indicator of transmural necrotic segments in the acute phase, while circumferential or radial strain to a significant lesser extent develop abnormal systolic lengthening and radial thinning despite transmural necrosis.

The relatively high number of transmural necrotic segments exhibiting positive peak longitudinal strain values when compared with circumferential strain and radial strain may reflect differences in wall stress sustained by the myocardium in long and short axis. According to the law of Laplace, local wall stress increases with increasing radius of the curvature. Longitudinal fibres, might therefore be exposed to higher wall stress compared to the wall stress generated by the relatively smaller radii of the short-axis curvatures, which are relevant for circumferential and radial strain [28]. Thus, differences in wall stress due to differing wall curvatures between longitudinal, radial and circumferential axes may possibly explain the more frequently observed abnormal systolic lengthening for longitudinal strain, when compared with circumferential strain and radial strain. Even though the contractile state of all fibres in transmural necrotic myocardium is the same, tethering and smaller wall curvatures prohibit regional stretching in the circumferential and radial direction.

### Limitations of the study

The pig as a research animal is a limitation to this study. The porcine heart’s poor tolerance to ischemia restricted us to induce relatively small infarctions, which resulted in the vast majority of the transmural necrotic segments being located in the apex. Although our results indicate that peak longitudinal strain better separates transmural necrotic segments from predominantly viable segments than circumferential strain and radial strain in acute ischemia, more research is needed to establish whether this is true also for larger infarctions and in the human heart. On the other hand, 2D-STE might be of more benefit to those patients suffering from ischemia with small infarctions, than patients with large infarctions and obvious signs of extensive myocardial ischemia, such as ST-elevation.

The 18-segment model in the present study with six apical segments also makes it difficult to directly compare our results with other studies which have employed 16-segment models. Since the 18-segment model consists of two more apical segments than the 16-segment model, it may be argued that the two extra apical segments are decisive for our results. To examine whether our results would change with four segments in the apex, a modified 16-segment model was created by averaging strain values from both septal and lateral segments. Measurements of necrosis was then recalculated for the two 120° apical septal and apical lateral segments, yielding a modified 16-segment apical model with 4 segments in the apex (2x60° + 2x120°). When ROC curve analysis was performed on this modified 16-segment model, the area under the curve for longitudinal strain was still found to be significantly larger than for circumferential strain and radial strain, for separating transmural necrotic segments from predominantly viable segments.

## Conclusions

We conclude that 2D-STE provides reliable and reproducible estimates of the extent of segmental necrosis. By measuring peak positive or negative strains during ejection time, our data suggest that longitudinal strain provides the most accurate estimate of the degree of transmurality in an acute ischemia-reperfusion model.

## Abbreviations

AUC: Area under the curve; LAD: Left anterior descending coronary artery; MI: Myocardial infarction; ROC: Receiver operator characteristic; 2D-STE: Two-dimensional speckle tracking echocardiography; TTC: 2,3,5-triphenyltetrazolium chloride.

## Competing interests

The author(s) declare that they have no competing interests.

## Authors’ contributions

The authors contributions were as follows: EA, ES and RB designed the study. EA and ES performed the experiments. AR obtained all echocardiographic images. EA and AR performed the echocardiographic analysis. EA carried out statistics and wrote the first manuscript draft. AR, RB and ES were all involved in the revision of the first manuscript draft. All authors read and approved the final manuscript.
